# Southern Surgical and Gynæcological Association

**Published:** 1893-11

**Authors:** 


### THE SOUTHERN SURGICAL AND GYNECOLOGICAL ASSOCIATION.

---

PROGRAMME OF THE SIXTH ANNUAL MEETING TO BE HELD IN NEW ORLEANS, LOUISIANA, ON NOVEMBER 14TH, 15TH AND 16TH, 1893.

---

ADDRESS OF WELCOME AND RESPONSE.

1. Memorial Address on Dr. Ephraim McDowell.—By L. S. McMurtry, M. D., Louisville, Ky.

2. Diagnosis of Pelvic Inflammatory Diseases.—By Howard A. Kelly, M. D., Baltimore, Md.

3. The Treatment of Pyo Salpinx.—By Cornelius Kollock, M. D., Cheraw, S. C.

4. The Incision in Abdominal Section—How to Close it—Post Operative Complications about it.—By Joseph Price, M. D., Philadelphia, Pa.

5. Aetiology and Treatmant of Post Operative Ventral Hernia.—By Chas. A. L. Reed, M. D., Cincinnati, Ohio.

6. The Vaginal Method as Compared with the Abdominal in Operations on the Uterus and its Appendages.—By Geo. J. Engelmann, M. D., St. Louis, Mo.

7. The Best Time to Operate in Appendicitis.—A. M. Cartledge, M. D., Louisville, Ky.

8. An Extra Peritoneal Method of Operating in Certain Cases of Appendicitis.—By Bacon Saunders, M. D., Ft. Worth, Texas.

9. Intra-Cranial Neurectomy and Removal of the Gasserian Ganglion, with Report of Cases.—By Louis McLane Tiffany, M. D., Baltimore, Md.

10. Contusion of the Brain.—By J. B. Murfree, M. D., Murfreesboro, Tenn.

11. Trephining as a Cure for Traumatic Epilepsy.—By Jno. T. Chapman, M. D., Bessemer, Ala.

12. Some Remarks on the Treatment of Epilepsy.—By B. E. Hadra, M. D., Galveston, Texas.

13. A Contribution to the Study of the Prostate.—By G. Frank Lydston, M. D., Chicago, Ill.

14. The Management of the Epicystic Fistula in Cases of Enlarged Prostate.—By J. D. S. Davis, M. D., Birmingham, Ala.

15. Choice of Operation for Stone in the Urinary Bladder. —By W. T. Briggs, M. D., Nashville, Tenn.

16. Supra Pubic Cystotomy.—By B. W. Taylor, M. D., Columbia, S. C.

17. Conditions Modifying the Prognosis in Gunshot Wounds of the Abdomen.—By A. B. Miles, M. D., New Orleans, La.

18. Treatment of Gunshot Wounds, with Report of Cases. —By W. F. Westmoreland, M. D., Atlanta, Ga.

19. How to Deal with Gunshot Wounds Safely without Delay.—W. L. Robinson, M. D., Danville, Va.

20. Laparotomy in General Surgery—Report of Twenty-two Cases.—By W. B. Rogers, M. D., Memphis, Tenn.

21. Celiotomy, with Report of Cases.—W. H. Wathen, M. D., Louisville, Ky.

22. Hypertrophy of the Omentum in Hernia, with Specimen.—By G. A. Baxter, M. D., Chattanooga, Tenn.

23. Wyeth's Bloodless Method in Amputation at Hip.—By F. W. Parham, M. D. New Orleans, La.

24. The Southern Surgical and Gynecological Association. Its Origin, Objects and Aims.—By Bedford Brown, M. D., Alexandria, Va.

25. Cancer; Its Etiology and Treatment.—W. L. Rodman, M. D., Louisville, Ky.

26. Operative Procedure for Cancinomatous Tumors of the Breast.—By J. McFadden Gaston, M. D., Atlanta, Ga.

27. Wounds of the Bladder, their Recognition and Management.—By Richard Douglas, M. D., Nashville, Tenn.

28. Fibroid Tumors Complicated with Pregnancy.—W. D. Haggard, M. D., Nashville, Tenn.

29. The Diagnosis of Some Abdominal Tumors Supposed to be Ovarian.—By Jas. A. Goggans, M. D., Alexandder City, Ala.

30. Rhinoplastic Operations.—By W. S. Elkin, M. D., Atlanta, Ga.

31. Sarcomata of the Peripheral Nerves, with Report of Case.—By W. O. Roberts, M. D., Louisville, Ky.

32. Division of the Cervix Uteri.—By H. P. C. Wilson, M. D., Baltimore, Md.

33. Some Experiments with the Galvanic Current on the Endometrium.—By H. Berlin, M. D., Chattanooga, Tenn.

34. A Combination of Carbolic Acid and Camphor as an Antiseptic.—By Wm. Perrin Nicolson, M. D. Atlanta, Ga.

35 A Case of Popliteal Aneurism.—By R. M. Cunningham, M. D., Pratt City, Ala.

36. The present Status of Ureteral Surgery.—By Arch. Dixon, M. D., Henderson, Ky.

37. Operative Procedure for Calculi in the Pelvis of the Kidney.—By W. F. B. Davis, M. D., Birmingham, Ala.

38. Two Unique Cases in Abdominal Surgery and Obstetrics.—By K. P. Moore, M. D., Macon, Ga.

39. Four Cases of Hysterectomy by Baer's Method.—By Jos. Taber Johnson, M. D., Washington, D. C.

40. Placenta Previa.—By George Ross, M. D., Richmond, Va.

41. Some Interesting Cases from Surgical Practice.—By F. W. Parham, New Orleans, La.

42. Report of Surgical Cases.—By Wm. H. H. Cobb, M. D., Goldsboro, N. C.

43. Treatment of Stone in the Ductus Communis Choledocus.—By Wm. E. B. Davis, M. D., Birmingham, Ala.

